# The future of health in Zimbabwe

**DOI:** 10.1080/16549716.2018.1496888

**Published:** 2018-07-30

**Authors:** Khameer K. Kidia

**Affiliations:** Kushinga, Harare, Zimbabwe

**Keywords:** Zimbabwe, health, Mugabe

## Abstract

In November 2017, following a military intervention, Robert Mugabe was forced to resign as president of Zimbabwe – where he had ruthlessly ruled since 1980. Mugabe’s regime was responsible for destroying the country’s excellent health system. I argue that this is a unique moment for health reform in Zimbabwe. This reform should focus on three areas: (1) repairing relationships with the international community by focusing on human rights and eliminating corruption, (2) strengthening the health workforce through retention strategies, training, and non-specialist providers, and (3) community engagement. The future of Zimbabwe’s health system is in limbo, and now is a unique opportunity to make positive change.

We are at a critical juncture for the future of Zimbabwe’s health. Robert Mugabe’s authoritarian regime, notorious for political repression and for an inflation rate of 79.6 billion percent in 2008 [1], also destroyed one of Africa’s most robust healthcare systems. In the wake of a military intervention resulting in the resignation of the 93-year-old Zimbabwean leader and assumption of office by new president Emmerson Mnangagwa, the current moment is a window for political and economic reform, but also for those working in the health sector to start rebuilding.

Historically, Zimbabwe boasted a thriving teaching hospital network, a strong primary healthcare system installed in the 1980s by the Mugabe regime [2], and a motivated, highly trained health workforce [3]. However, during his reign, Mugabe also undermined this health system through human rights abuses and economic mismanagement, among other actions. In order to maintain his grip on power, Mugabe incited horrific violence against members of the opposition, inflicting psychological and physical trauma on Zimbabweans [4–6]. His party harassed doctors and nurses who cared for the victims [6]. The former president further politicized health by denying the existence of a severe cholera epidemic in 2008, despite the death of 4000 people [7–9].

In the 2000s, the economy was collapsing under the weight of debt and corruption, which led to infrastructural decay and a lack of basic health supplies. Government spending on health dropped from 7% in 2000 to 4% in 2007 [10]. Some hospitals did not have electricity or running water, let alone scalpels or painkillers. The Ministry of Health could no longer afford to pay its health workers. In 2008, government physicians earned less than US$1 per month [6]. Abysmal working conditions drove 20% of these health professionals abroad each year [6]. In the face of global health threats – notably HIV/AIDS, which struck Zimbabwe harder than nearly any other country – the health sector was left defenseless.

Yet the broken health system in Zimbabwe retains its original foundations, making it relatively robust compared to other sub-Saharan African countries. The country’s success in tackling the HIV/AIDS epidemic is a case in point. Despite a harsh political and economic climate, Zimbabwe reduced HIV prevalence from 29% in 1997 to 16% in 2007, largely due to community awareness and behavior change [11,12]. This demonstrated community resilience makes it possible for us to rebuild and strengthen the health system rather than starting from scratch. Political transitions can be moments of significant institutional reform – political norms are in flux, and there are injections of aid, new political alliances, and renewed optimism [13]. While the new government has allocated a disappointing 7.7% to health in the 2017–2018 national budget [10], during this political transition there are additional political and structural ways to strengthen the health system. To profit from this transition period, we should consider three approaches: repair relationships with the international community; strengthen the health workforce; and prioritize innovative community-based models of care.

## Improve diplomatic relations: focus on human rights and eliminating corruption

Now is the time to mend diplomatic relations. Zimbabwe has become a pariah state: isolated from the international community due to decades of corruption [14], political violence [5], and lack of transparency. In order to punish the Mugabe regime, the International Monetary Fund stopped lending to Zimbabwe in 1999 and a litany of sanctions followed, by the US, UK, and EU, making it difficult for aid organizations to deliver food and health aid and discouraging foreign direct investment [15–17]. Although foreign governments lifted some sanctions or imposed ‘targeted’ sanctions [18], more can be done. President Mnangagwa recently called for universal lifting of sanctions [19]. Although often designed to curtail human rights abuses, the effectiveness of sanctions is an ongoing debate [20–22]. Even ‘targeted’ sanctions have mixed results: they often do not produce policy change on the ground [18], and in Zimbabwe have had the knock-on effect of decreasing donor and investor willingness to engage. Ultimately, these sanctions end up punishing some of the most vulnerable citizens rather than perpetrators of human rights abuses.

To reconcile these broken relationships, Zimbabwe needs, among other things, to re-establish its legitimacy by prioritizing a human rights approach to health. Refocusing the public health debate on human rights has been a successful strategy of organizations such as Partners in Health in places such as Haiti, Malawi, and Liberia [23]. In Zimbabwe, a human rights approach should include structural access to clean water, food, and humane living conditions; protection for vulnerable populations; and a zero-tolerance policy for political violence and intimidation. The government must also fight corruption and cronyism in the health sector by working with international organizations to create strong accountability mechanisms at both the national and district levels. This will ensure that donor aid and local funding reaches intended communities.

In the same vein, Western governments need to continue to cautiously lift sanctions, especially those that disproportionately harm vulnerable citizens. During this process, the international community, especially Britain, needs to be cognizant of Zimbabwe’s colonial legacy. Respecting sovereignty is a major challenge in global health governance and it will be paramount for health reform in Zimbabwe [24]. Although multiple economists argue that aid altogether is deleterious to African countries because it fuels corruption and creates dependent states [25,26], we should interpret these data cautiously when human lives are on the line. In 2012, official development aid accounted for 60% of Zimbabwe’s health financing [27]. Without this assistance, even basic health resources would be limited. More problematic than aid itself are overly vertical programs that do not reach beyond specific, disease-focused interventions [28–30]. Health initiatives – in order to be sensitive to concerns of sovereignty, dependency, and sustainability – must not be viewed by donors as business opportunities, laboratories for drug trials, or piecemeal interventions that briefly alleviate a single issue. The priority should be on funding practical health programs and civil society, with a long-term focus to rebuild an integrated, stronger health system. This is achievable in Zimbabwe because there is an existing skeleton system that can be used as a platform for integrating health services across the country.

## Strengthen the health workforce

In the 1990s and 2000s, many of Zimbabwe’s health professionals fled to neighboring sub-Saharan African countries and to the UK to seek better pay and higher living standards [6]. The Zimbabwean government’s response was a partnership with Cuba to deploy hundreds of Cuban physicians within the Zimbabwean health system, together with a series of measures, such as bonding newly trained health workers and improved training [3]. With fairly strong training programs for nurses and physicians already in place, the focus should shift to retention of newly trained health professionals through fair pay and non-monetary incentives such as supportive work environments, physical safety, and leadership opportunities [31].

Some innovative ways to strengthen the health workforce would be: (1) to encourage retention and motivation of current workers through better, safer work environments; (2) to build leadership capacity in the workforce, potentially by mobilizing the Zimbabwean health diaspora to engage in training and skill-building among the current workforce [3]; and (3) to place a greater reliance on mid-level and non-specialist providers [32] and on community members including community health workers (CHWs) [33–36] and traditional healers [37–40].

## Prioritize communities

In Zimbabwe, the majority of the population live in rural areas, where people have the greatest need for and least access to health services [27]. To effect meaningful change that does not depend on donor aid or highly skilled workers, policymakers, funders, and implementers should prioritize community engagement, i.e. empowering citizens to promote and deliver healthcare in their own areas by focusing on ‘local ideas, concerns, and opportunities’ [41,42]. This will mean hiring Zimbabweans, building capacity, and listening carefully to local priorities. Community health models, often using community health workers, have been successful in many areas of global health including HIV/AIDS, child health, malaria, and tuberculosis [33,35,36]. Although a village health worker program was installed in the 1980s via the Health Transition Fund, this effort was limited by a lack of true community participation and a bureaucratic top-down approach where decisions were still made by the Ministry of Health [2]. In re-envisioning community engagement, more radical approaches, which truly provide agency to local actors, are needed. A successful Zimbabwean example, the Friendship Bench, already exists for mental health. In this model, lay community members are trained to deliver basic cognitive behavioral therapy on a bench outside district clinics in Zimbabwe. The intervention has been a triumph and is shown in a clinical trial to significantly reduce rates of depression and common mental disorders [43]. The intervention further engages the community by working explicitly with district health offices (rather than national-level Ministry of Health departments); it also includes income-generating activities that promote financial independence among patients, and community awareness initiatives. It is being scaled countrywide and integrated into rural health systems with careful cultural and linguistic adaptation. This type of innovation, around low-intensity, community-based care, should become the cornerstone of healthcare reform in Zimbabwe.

Authoritarian regimes have a tendency to centralize power. In Zimbabwe’s current system, most strategic health decisions are made at the national level rather than in district health offices. Innovative community health interventions such as the Friendship Bench would help to spread decision-making authority and flatten the current hierarchy in a more purposeful way than prior attempts. This approach would grow local leadership and ensure that interventions are socially and culturally sensitive. By giving the local community agency in setting health priorities, there would also be smaller budgets, built-in accountability, and less room for corruption. Finally, community health interventions do not require large infrastructural investment or lengthy training programs. Instead, they need strong social engagement and political will. A major political transition, such as the one Zimbabwe is currently experiencing, is the perfect time to garner this type of energy.

Although the fall of a dictator does not necessarily imply a prompt return to democracy, political transitions are critical moments shared by several low- and middle- income countries, many of which have similar challenges to Zimbabwe in healthcare equity. Galvanizing local and global health communities to take action during these times has the potential for reprioritization of health and even pivotal healthcare reform. In an age of competitive global resources, these are opportunities that we cannot afford to miss.

## Paper context

In November 2017, following a military intervention, Robert Mugabe was forced to resign as president of Zimbabwe – where he had ruthlessly ruled since 1980. Mugabe’s regime was responsible for destroying the country’s excellent health system. This piece argues that this is a unique moment for health reform in Zimbabwe. This reform should focus on three areas: (1) repairing relationships with the international community, (2) strengthening the health workforce, and (3) community-based care.
